# Optimizing bone wound healing using BMP2 with absorbable collagen sponge and Talymed nanofiber scaffold

**DOI:** 10.1186/s12967-018-1697-y

**Published:** 2018-11-21

**Authors:** Emily L. Durham, R. Nicole Howie, SarahRose Hall, Nicholas Larson, Brayden Oakes, Reed Houck, Zachary Grey, Martin Steed, Amanda C. LaRue, Robin Muise-Helmericks, James Cray

**Affiliations:** 1 Department of Oral Health Sciences, Medical University of South Carolina, 173 Ashley Ave., Charleston, SC 29425 USA; 2 College of Dental Medicine, Medical University of South Carolina, 173 Ashley Ave., Charleston, SC 29425 USA; 3 Department of Oral and Maxillofacial Surgery, Medical University of South, 173 Ashley Ave., Charleston, SC 29425 USA; 4 Department of Pathology and Laboratory Medicine, Medical University of South, 173 Ashley Ave., Charleston, SC 29425 USA; 5 Ralph H. Johnson Veterans Administration Medical Center, 109 Bee St, Charleston, SC 29401 USA; 6 Department of Regenerative Medicine, Medical University of South Carolina, 173 Ashley Ave., Charleston, SC 29425 USA; 7 Division of Anatomy, Department of Biomedical Education & Anatomy, The Ohio State University College of Medicine, 279 Hamilton Hall, 1645 Neil Ave., Columbus, OH 43210 USA

**Keywords:** BMP2, Bone wound healing, Biomaterials

## Abstract

**Background:**

Bone is a highly vascularized and resilient organ with innate healing abilities, however some bone injuries overwhelm these attributes and require intervention, such as bone tissue engineering strategies. Combining biomaterials and growth factors, such as bone morphogenetic protein 2 (BMP2), is one of the most commonly used tissue engineering strategies. However, use of BMP2 has been correlated with negative clinical outcomes including aberrant inflammatory response, poor quality bone, and ectopic bone.

**Methods:**

In the present study, a novel poly-*n*-acetyl glucosamine (pGlcNAc, trade name Talymed) scaffold was utilized in addition to the commonly used acellular collagen sponge (ACS) BMP2 delivery system in a murine calvarial defect model to investigate whether the innate properties of Talymed can reduce the noted negative bone phenotypes associated with BMP2 treatment.

**Results:**

Comparison of murine calvarial defect healing between ACS with and without Talymed revealed that there was no measurable healing benefit for the combined treatment. Healing was most effective utilizing the traditional acellular collagen sponge with a reduced dose of BMP2.

**Conclusions:**

The results of this investigation lead to the conclusion that excessive dosing of BMP2 may be responsible for the negative clinical side effects observed with this bone tissue engineering strategy. Rather than augmenting the currently used ACS BMP2 bone wound healing strategy with an additional anti-inflammatory scaffold, reducing the dose of BMP2 used in the traditional delivery system results in optimal healing without the published negative side effects of BMP2 treatment.

## Background

Bone is a highly vascularized and resilient organ with innate healing abilities, however some bone injuries overwhelm these attributes and thus require significant medical intervention [1]. Therapeutic strategies used to treat such cases include the use of grafts [2], implantable medical devices such as plates and pins, and tissue engineering strategies. Autologous bone grafting remains the standard of care for bone wound healing due to its consistent clinical outcomes [2] however, its use is severely hampered by short supply and considerable donor site morbidity associated with the harvest [3, 4]. Thus, bone tissue engineering strategies are needed.

Currently bone tissue engineering uses cell therapy, biomaterials/scaffolds, and/or manipulation of osteogenic signaling pathways to overcome failures in the treatment of bone wounds [5–9]. Combining biomaterials and growth factors such as bone morphogenetic protein 2 (BMP2) is one of the most commonly used tissue engineering strategies in orthopedic and craniofacial surgery [10, 11]. Despite the clinical efficacy of BMP2 in bone regeneration, adverse clinical events such as extreme inflammation and edema, as well as poor quality bone regenerate and uncontrolled ectopic bone formation necessitate optimization of the application of this highly effective osteogenic factor [12–15]. The current United States-Food and Drug Administration approved scaffold for BMP2 delivery is absorbable collagen sponge (ACS; INFUSE Bone Graft, Medtronic TM), however use of this scaffold peptide combination has been linked to aberrant inflammatory responses precipitating poor quality bone and ectopic bone growth [8, 9, 16–19].

ACS releases BMP2 at a rate that is unable to sustain quality bone growth [9, 20]. Controlling the release of BMP2 from the scaffold may allow for decreased dosing and promote an extended period of efficacy while eliminating the negative side effects of this engineering strategy. The United States-Food and Drug Administration approved pGlcNAc nanofiber scaffold (Talymed; Marine Polymer Technologies, Danvers, MA) has been vetted for augmentation of wound healing and offers extended BMP2 retention [9, 21–24]. This biomaterial is a polysaccharide derived from a marine diatom that has been shown to reduce scar formation, allow for increased alignment of collagen fibers, increased collagen tensile strength, and reduced inflammatory response in addition to extended retention of BMP2 [9, 25].

In the present study, the novel Talymed scaffold was utilized in addition to the approved ACS BMP2 delivery system to investigate whether the innate properties of Talymed can reduce negative outcomes associated with the current BMP2 delivery system. Due to the enhanced BMP2 binding and anti-inflammatory qualities of Talymed, we hypothesize that bone regenerated using combined ACS/Talymed as a BMP2 delivery system will yield better healing outcomes as compared to the current clinical standard scaffold (ACS) alone.

## Materials and methods

### Animals and treatments

**Fig. 1:**
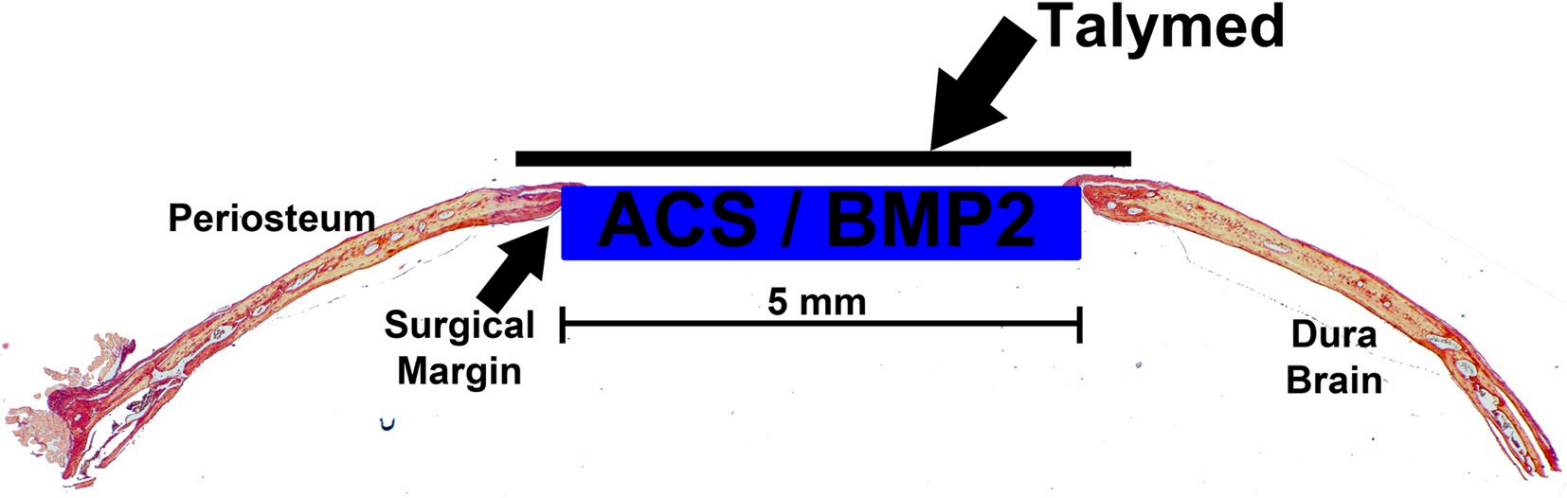
Methodological schematic of ACS/Talymed Scaffold. Representative histological section of a 5 mm defect implanted with ACS soak loaded with BMP2 and overlaid with Talymed. Note the orientation with the Talymed replacing the removed periosteum over the defect

Adult (8 week old) male and female C57BL6 mice (n = 80) (Jackson Laboratory, Bar Harbor, ME) were randomized into one of eight surgical groups implanted with ACS alone or ACS overlaid with Talymed (ACS/Talymed) matrix soak loaded with control (sterile H_2_O), medium (325 ng), high (542 ng) or scaled clinical (5000 ng) doses of BMP2 (Table 1, Fig. 1). Under sterile conditions, 6 mm biopsy punches of ACS and 5 mm biopsy punches of Talymed were obtained and stored in a 24 well plate lined with sterile gauze. Fifteen minutes prior to implantation, matrices were loaded with BMP2 resuspended in 25 µl sterile water (Infuse, Medtronic, Memphis, TN) [9, 26]. One Small Kit of Infuse Bone Graft (Medtronic) which includes sterile water, sterile BMP2, and sterile absorbable collagen sponge (ACS) was used to complete this entire investigation.

**Table 1:** Sample sizes of C57BL/6J mice for the associated BMP2 doses and scaffolds

Time point	ACS				ACS/Talymed			
	Control	Medium BMP2	High BMP2	Clinical BMP2	Control	Medium BMP2	High BMP2	Clinical BMP2
4 weeks	10 (5M, 5F)	10 (5M, 5F)	9 (4M, 5F)	9 (4M, 5F)	10 (5M, 5F)	10 (5M, 5F)	10 (5M, 5F)	10 (5M, 5F)

### Critical-sized calvarial defect model

For each specimen, a critical-sized calvarial defect was performed as previously described [8, 9, 26–28]. Briefly, the mice were anesthetized with isoflurane (Bethlehem, PA, USA) and a midline scalp incision was used to expose and remove the periosteum. A 5-mm round craniectomy defect was created using a slow-speed hand drill. After the application of experimental treatment, the incision was closed with 6 × 0 polypropylene suture (removed 2 weeks later). Animals were weighed and monitored 2 days post-surgery for any signs of pain or distress. Daily monitoring continued until time of sacrifice (4 weeks) when mice were euthanized by asphyxiation using CO_2_ gas followed by secondary cervical dislocation. Skulls were collected for downstream analyses. All procedures were carried out with the approval of the Medical University of South Carolina IACUC (AR #3452), in an Association for Assessment and Accreditation of Laboratory Animal Care International accredited facility where all husbandry and related services were provided by the Division of Laboratory Animal Resources. The animals were housed 5 to a cage which contained enrichment material and were allowed access to food and water ad libitum. All procedures and the reporting thereof comply with the Animal Research: Reporting in Vivo Experiments (ARRIVE) guidelines [29].

### Micro-computed tomography (µCT) image analysis of bone regeneration

Collected skulls were removed of soft tissue and bisected from the occipital protuberance to nasale in the transaxial plane. Micro-computed tomography (µCT) images of the defect areas were obtained via ex vivo Skyscan 1176 (Skyscan, Aartlesaar, Belgium) with a 0.5 mm thick aluminum filter at a voltage of 50 kV and current of 500 µA. Data were acquired at an isotropic resolution of 18 µm, rotation step of 0.5°, and 180° rotation. Reconstruction and analyses were performed on the blinded samples using NRecon and CTAn SkyScan software. A global threshold of 100–255 was used. Standard 3D morphometric parameters [30] were determined for the region of interest (ROI; 5.0 mm circle, 51 cuts equaling 918 µm), positioned along the initial surgical margins for all samples. A secondary region of interest encompassing all ectopic bone for each sample was also analyzed for standard morphometric parameters. Representative 3D images were created using CTvol software.

### Tissue processing and staining

Blinded, representative samples (n = 4) from each group were bisected in the coronal plane through the center of the surgical defect and prepared for paraffin sectioning by fixing in 3.7% formaldehyde for 2 days. Subsequently, samples were decalcified in 0.25 M EDTA at pH 7.4 for 21 days and then washed, dehydrated in graded ethanol (70–100%), cleared in xylene, and embedded in paraffin. Histology was performed on 3, 7 µm sections in the coronal plane at least 40 μm apart per sample for analysis of the defect area. Standard procedures were employed for hematoxylin and eosin, Masson’s trichrome (Thermo Scientific, Waltham, MA USA), and picro sirius red (non-polarized and polarized) staining [8, 9]. Stained sections were photographed using a Motic Inverted Microscope with attached camera (Motic, British Columbia, Canada) and quantified using NIH Image J and Visiopharm (Visiopharm, Broomfield, Colorado) software. For analysis, a region of interest including the surgical margins but excluding ectopic bone growth above the native bone along the surgical plane was isolated. Nuclei within the defects were quantified using color deconvolution and creation of masks to count darkly stained nuclei. Total area of bone (osteoid) was analyzed for Mason’s trichrome, and picro sirius red sections were analyzed for total collagen area (non-polarized), thin immature (green; polarized), intermediate (yellow; polarized), and thick mature (red; polarized) collagen fibers.

### Statistical analysis

Two-way ANOVA Scaffold x Treatment with post hoc Bonferroni analyses were conducted for all comparisons where appropriate. Transformations of the data were performed where needed to correct for violations and meet the assumptions of homogeneity of variance or normality. Non-parametric analyses were utilized where necessary for unavoidable violations of assumptions. Differences were considered significant if p ≤ 0.05. Data are presented as mean ± SEM. A power study for experimentation was calculated based on an α = 0.05, β = 0.8, η > 0.4, necessitating 10 animals per group. *p ≤ 0.05, **p ≤ 0.01, and ***p ≤ 0.001.

## Results

**Fig. 2:**
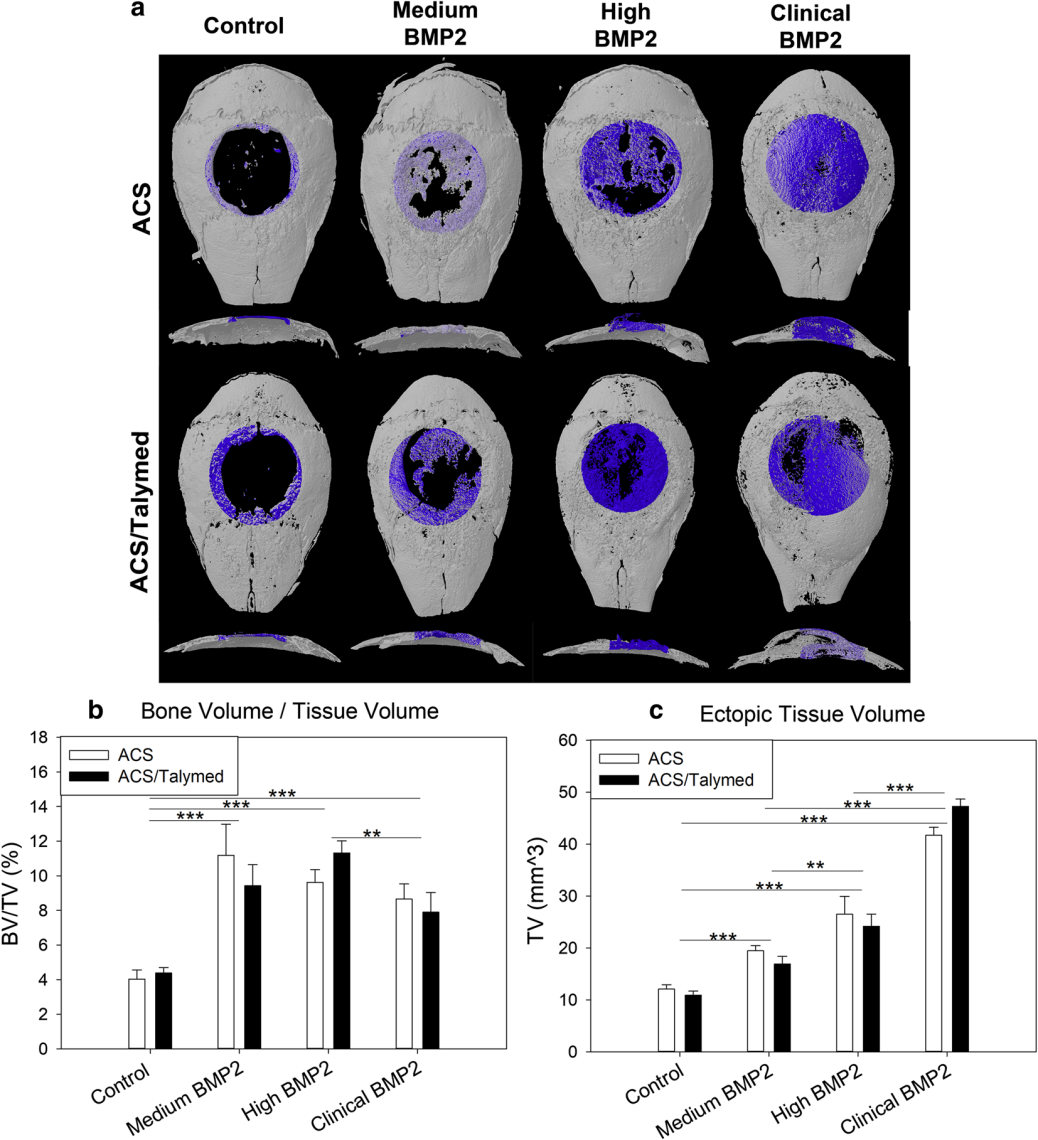
µCT assessment of bone healing. **a** Representative µCT reconstructions of 4 week 5 mm critical sized calvarial defect healing from above (above) and in cross-section (below) for both ACS (top) and ACS/Talymed (bottom) treated individuals. Healing in the defect area has been highlighted and BMP2 dose increases from left to right. Note the similar healing between medium and high doses of BMP2 and the dramatic ectopic bone highlighted in the clinical dose of BMP2. **b** Assessment of bone volume/tissue volume confirms greater healing with medium and high doses of BMP2 and less healing with the clinical dose. **c** A secondary analysis of the ectopic bone not included within the surgical plane confirmed a dose dependent increase in aberrant bone. n = 9 or 10 per group **p ≤ 0.01, ***p ≤ 0.001

Analysis of healing within the calvarial defects using standard morphometric measures of µCT scans revealed no difference between the ACS alone and ACS combined with the novel Talymed scaffold (ACS/Talymed) (Fig. 2a, b). The dose of BMP2 did however, induce increased healing with all doses precipitating significantly more bone than the control (p < 0.001). The most concentrated dose of BMP2 (clinical) did not yield the greatest amount of healing (p = 0.005 compared to high BMP2) but did precipitate the greatest amount of ectopic tissue volume (p < 0.001). Ectopic tissue volume increased with BMP2 dose. The medium dose presented less ectopic bone than both high (p = 0.003) and clinical (p < 0.001) doses, but more than control (p < 0.001) (Fig. 2c).

**Fig. 3:**
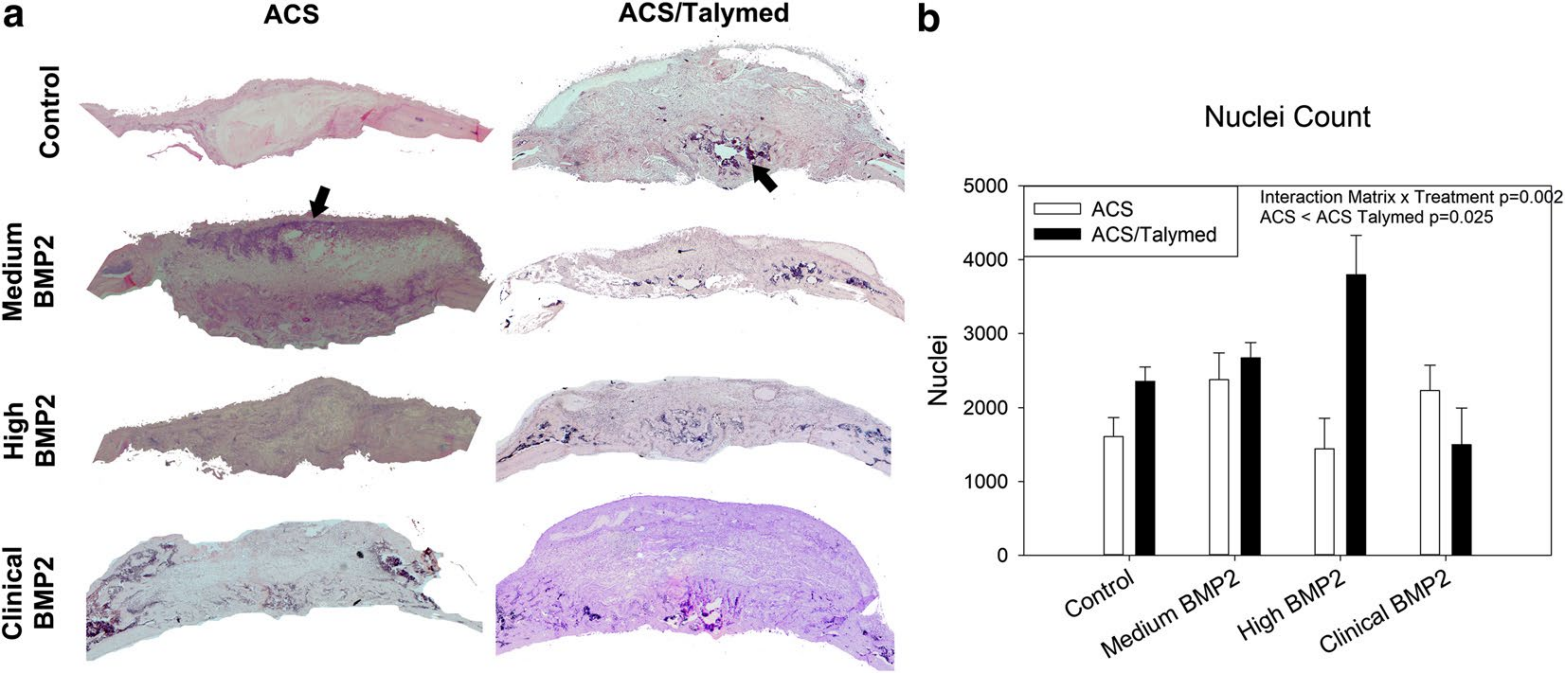
Assessment of cell presence in healing defects. **a** Representative hematoxylin and eosin stained healing defects with ACS (left) and ACS/Talymed (right). BMP2 dose increases from top to bottom. Arrows indicate highly cellular areas. 10× magnification. **b** Quantification of nuclei within the defect area indicates that more cells are present in the ACS/Talymed treated defects however there is an interaction effect highlighted by fewer cells in each scaffold with the clinical dose of BMP2. n = 3 sections 40 µm apart from at least four individuals per group

Histological staining of the defects allowed for a more specific assessment of the healing process. Observation of the morphology via hematoxylin and eosin staining of the defect areas for the ACS/Talymed group indicates areas devoid of cells, and areas that lack the organization associated with both scar and bone (Fig. 3a). As new cell infiltration is vital for healing, an assessment of the number of nuclei within each defect site was performed. Absolutely more cells were present in the ACS/Talymed combined scaffolds than the ACS alone (p = 0.025). In correlation with the reduction in healing with the clinical dose of BMP2, cells within the defect are reduced with higher BMP2 dosages (Fig. 3b).

**Fig. 4:**
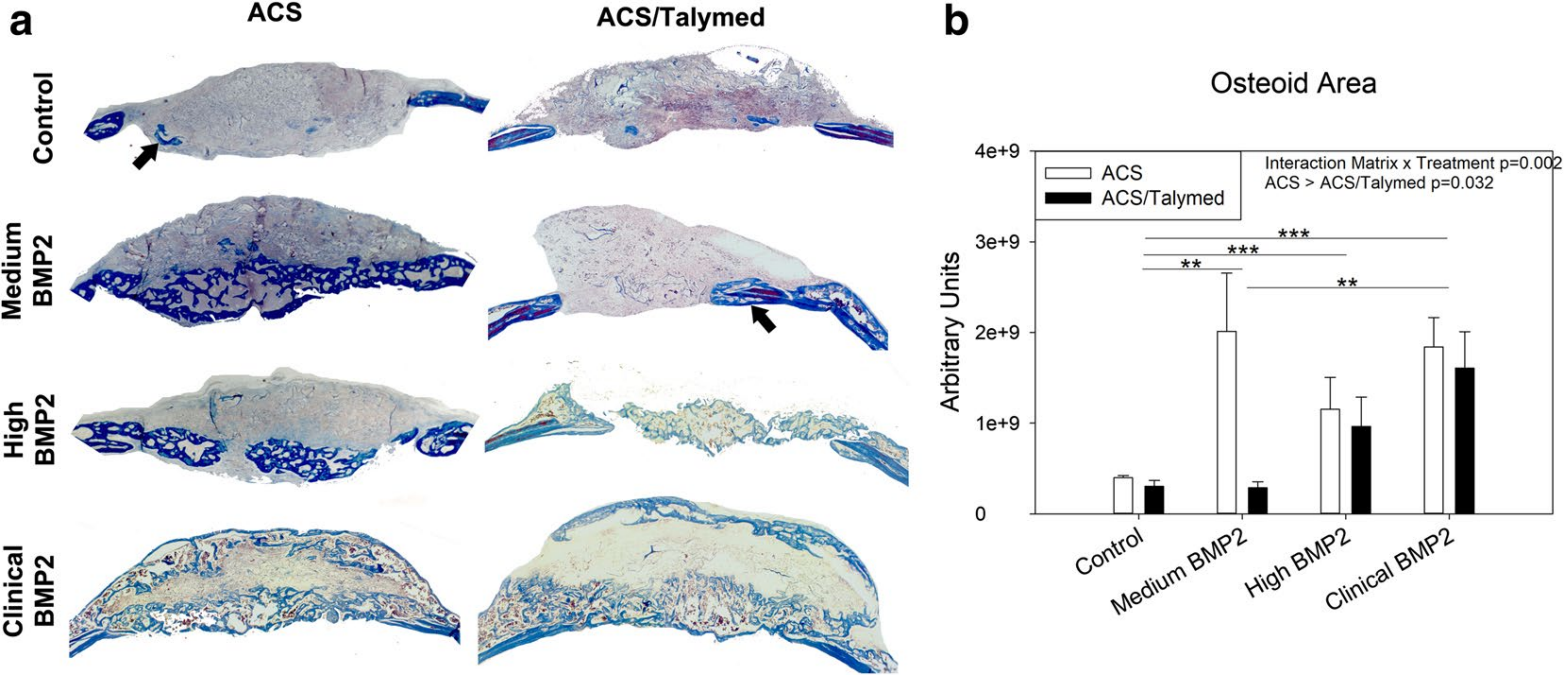
Assessment of osteoid tissue within healing defects. **a** Representative Mason’s Trichrome stained healing defects with ACS (left) and ACS/Talymed (right). BMP2 dose increases from top to bottom. Arrows indicate areas of osteoid tissue. Note greater density and organization of osteoid tissue in the ACS only specimen. 10× magnification. **b** Quantification of osteoid area indicates greater osseous healing with the ACS only scaffold and with BMP2 treatment. Note that the ACS with medium BMP2 precipitated the most osteoid tissue. n = 3 sections 40 µm apart from at least four individuals per group **p ≤ 0.01, ***p ≤ 0.001

Masson’s trichrome staining was used to quantify osseous healing within the defects. Assessment of the area of osteoid indicates that more healing occurred within the ACS only defects as compared to the ACS/Talymed defects (p = 0.032) (Fig. 4). The most osteoid tissue was found within defects treated with the medium dose of BMP2. The osteoid tissue in these samples is tightly packed together and abundant as compared to the defects treated with the clinical dose of BMP2 (p = 0.006). Little, if any, osseous tissue can be found within the control defects from either scaffold (p < 0.001) (Fig. 4a, b).

**Fig. 5:**
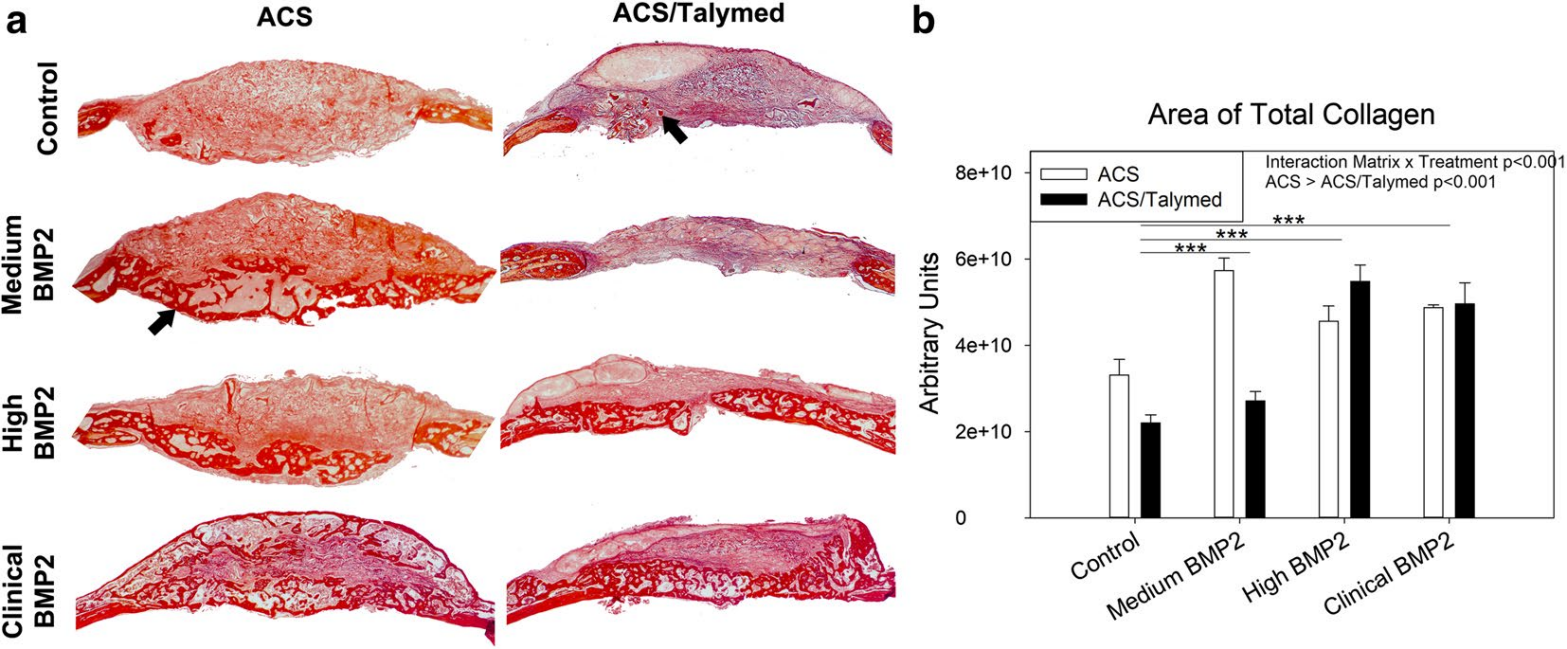
Assessment of total collagen within healing defects. **a** Representative picro sirius red stained healing defects with ACS (left) and ACS/Talymed (right). BMP2 dose increases from top to bottom. Arrows indicate areas of dense collagen. 10× magnification. **b** Quantification of total collagen within the defects indicates more collagen in the ACS only defects with collagen area increasing with BMP2 dose. Note that medium BMP2 with ACS only presents the greatest area of collagen. n = 3 sections 40 µm apart from at least four individuals per group ***p ≤ 0.001

**Fig. 6:**
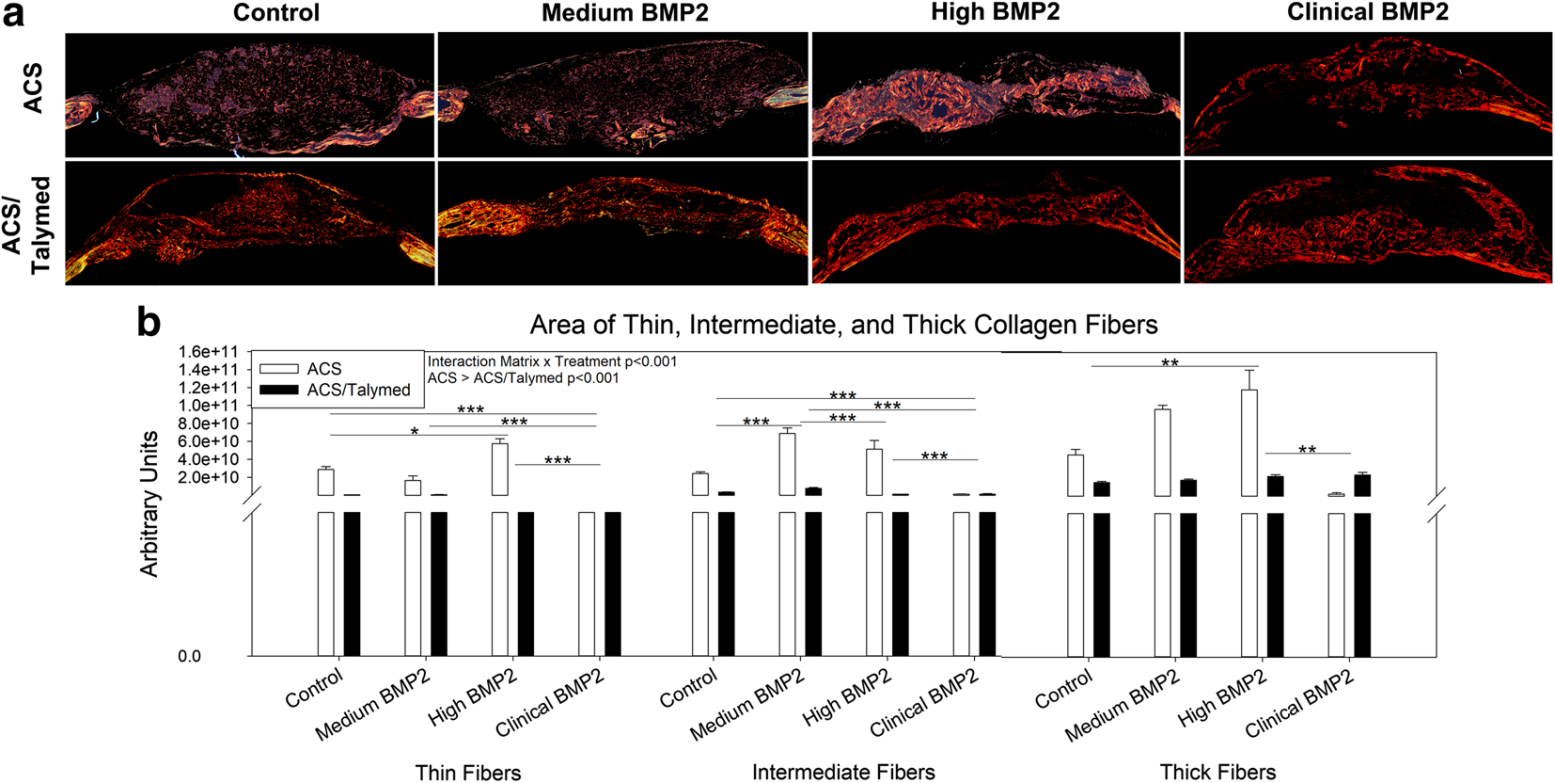
Assessment of collagen fiber maturity within healing defects. **a** Representative polarized picro sirius red stained healing defects with ACS (top) and ACS/Talymed (bottom). BMP2 dose increases from left to right. 10× magnification. **b** Quantification of thin (green) immature, intermediate (yellow), and thick (red) mature collagen fibers correlates with total collagen quantification with ACS presenting more of all fiber types as compared to ACS/Talymed. Note that the clinical dose of BMP2 presents the least of all fiber types while the other BMP2 doses present robust populations of fibers of all maturity levels indicating active turnover and remodeling. n = 3 sections 40 µm apart from at least four individuals per group *p ≤ 0.05, **p ≤ 0.01, ***p ≤ 0.001

Picro sirius red staining was used to assess collagen within the healing defects. A greater area of total collagen was found within the ACS only defects as compared to the defects filled with combined ACS/Talymed (p < 0.001). BMP2 treatment also precipitated a greater area of total collagen as compared to control (p ≤ 0.003). An interaction between the scaffold and the BMP2 treatment can be observed with the reduction of total collagen in the ACS only scaffolds with increasing BMP2. At the highest dosage of BMP2, the defect areas are disorganized in both scaffolds as compared to the dense nature of the tissue within the healing defects treated with the lower doses of BMP2 (Fig. 5a, b). Polarization of picro sirius red staining allows for an assessment of the maturity of collagen fibers types. In correlation with the greater amount of total collagen found in the ACS only defects as compared to the ACS/Talymed combined treatment, more of each fiber type (thin, intermediate, and thick) were found in the ACS only defects (p < 0.001) (Fig. 6a). An interaction between matrix and dose of BMP2 was observed with a significant decrease in all fiber types from the high to clinical doses (p ≤ 0.005). The medium dose of BMP2 resulted in the most intermediate collagen fibers (p < 0.001) while the highest dose of BMP2 (clinical) presented with the fewest of all collagen fiber types (Fig. 6b).

## Discussion

Our hypothesis that adding an overlay of Talymed to the currently employed ACS BMP2 delivery system as a means of reducing negative outcomes was refuted by our µCT analysis. The combined scaffold did not produce better healing or more bone fill than the traditional delivery system. This outcome agrees with previous studies that found that the osteoinductive BMP2 along with an osteoconductive matrix such as ACS precipitates the greatest amount of bone growth [16, 25, 27, 31, 32]. Though we hoped that as in previous studies Talymed would precipitate denser bone growth, this did not occur when it was used with the ACS scaffold wounds [8, 9]. However, interestingly our lowest dose of BMP2 (medium) did provide the greatest amount of healing with the least amount of ectopic bone. The higher bone volume observed in the medium BMP2 ACS only group indicates that a lower dose of BMP2 may be most effective at healing bone wounds.

The importance of BMP2 dose was highlighted in our more specific histological assessment of healing. Despite the noted large acellular areas, there was greater cellular infiltration in the combined ACS/Talymed group. Though Talymed has been shown to augment healing in cutaneous wounds by enhancing cellular activity and possessing anti-inflammatory and non-immunogenic properties, these characteristics do not seem to translate into quality bone regeneration [9, 23, 24]. Assessment of the bone fill via Mason’s trichrome stain indicated that again the ACS without Talymed yields the greatest amount of bone fill. Further, the organization of the osteoid tissue, particularly in the ACS medium BMP2 specimen, best replicates osteoid tissue in the surrounding native bone. The osteoinductive nature of BMP2 can be readily noted with the lack of osteoid tissue in both the control groups; however, it is of note that there is less osteoid tissue with the highest (clinical) dose of BMP2 indicating that proper dosing of BMP2 is important for healing.

Assessment of the collagen within the healing defects again indicated the superiority of the ACS scaffold without the addition of Talymed. Not only did the ACS scaffold present the most total collagen, it also included the most of all types/maturity levels of collagen fibers. The lower doses of BMP2 included fibers of all maturity levels indicating areas of active turnover and remodeling. The clinical dose of BMP2, on the other hand, presented with the least amount of all fiber types including mature fibers. This may indicate that healing and turnover have ceased in these areas resulting in maintenance of the acellular space, ectopic bone, and a reduction in overall collagen matrix.

## Conclusion

Though Talymed can retain BMP2 better than ACS, this slower release may not be as important as BMP2 dose for healing [9, 12, 33]. Further, it appears as if the other positive attributes of Talymed including its anti-inflammatory and cell activity promoting characteristics, are not as effective at promoting quality bone wound healing when compared to a sub-clinical dosage of an osteoinductive peptide such as BMP2. Here we observed that healing was most effective with the traditional acellular collagen sponge and a reduced dose of BMP2 leading to the conclusion that excessive dosing of BMP2 may be responsible for the negative side effects observed with this bone tissue engineering strategy. Future studies will need to assess optimal dosing of BMP2 for bone wound healing as the socioeconomic burden of the 1 million fracture repair surgeries performed annually in the United States [34] clearly represent an unmet clinical need for improved bone tissue engineering strategies that promote bone healing.

## Abbreviations

BMP2: bone morphogenetic protein 2
pGlcNAc: poly-*n*-acetyl glucosamine, trade name Talymed
ACS: acellular collagen sponge
µCT: micro-computed tomography
